# Serological and Molecular Characterization of the Hepatitis B Virus in Blood Donors in Maputo City, Mozambique

**DOI:** 10.3390/v17010094

**Published:** 2025-01-13

**Authors:** Olga Maquessene, Osvaldo Laurindo, Lúcia Chambal, Nalia Ismael, Nédio Mabunda

**Affiliations:** 1 Instituto Nacional de Saúde of Mozambique, EN1, Bairro da Vila, Marracuene 3943, Mozambique; olga.maquessene@ins.gov.mz (O.M.); osvaldo.laurindo@ins.gov.mz (O.L.); nalia.ismael@ins.gov.mz (N.I.); 2 Faculty of Medicine, Universidade Eduardo Mondlane, Avenida Salvador Allende 1113, Maputo City 1100, Mozambique; luciachambal@gmail.com; 3 Hospital Central de Maputo, Avenida Agostinho Neto 164, Maputo City 1100, Mozambique

**Keywords:** HBV, blood donors, serological profile, genetic diversity, Mozambique

## Abstract

Hepatitis B virus (HBV) is a major public health concern responsible for hepatitis and hepatocellular carcinoma (HCC) worldwide. In Mozambique, HBsAg prevalence is high and endemic, and despite the strategies to mitigate the spread of the disease, the HCC incidence is still high and one of the highest in the world. There is still limited data on the serological profile and molecular epidemiology of HBV in Mozambique given the burden of this disease. In this study, we aimed to describe the serological and molecular characterization of HBV among blood donors. We conducted a cross-sectional survey from November 2014 to October 2015 at the Blood Bank of the Hospital Central de Maputo. Serological testing and molecular testing were performed. The frequency of HBV infection was estimated at 4.4% and was higher among males (79.1%), individuals aged 25–39 years (55.2%), and replacement donors (89.6%). The median viral load of HBV-positive blood donors was 1288.5 IU/mL, and 43.8% had a viral load higher than 2000 IU/mL. Most of the sequenced samples (94.3%) belonged to subgenotype A1. These findings underscore the importance of ongoing surveillance to inform effective HBV control strategies and present evidence about the burden of HBV among blood donors, which definitely requires attention, and clinical blood banks in Mozambique and in similar settings.

## 1. Introduction

In 2022, the World Health Organization (WHO) estimated that 254 million people worldwide were living with chronic hepatitis B virus (HBV), and between 2020 and 2022, the mortality rate mostly from cirrhosis and hepatocellular carcinoma (HCC) increased from 0.82 million to 1.10 million [1,2].

Nonetheless, the African region is the most affected, with an estimated HBV prevalence of 5.8% and an HCC incidence of 46,000/year [3,4]. For Mozambique, HBV is considered endemic with a prevalence of 8% and a incidence of HCC of 101 cases per 100,000 males per year, among the highest in the world [5,6,7]. Strategies to mitigate the burden of the disease as a public health concern include the accurate diagnosis and adequate treatment of infected individuals, prevention of mother-to-child transmission, vaccination at birth, and high-risk groups [8]. In 2022, it was estimated that globally only 13% of people living with chronic hepatitis B infection had been diagnosed and 3% received antiviral therapy, with Africa reporting the lowest rates [9,10]. Although routine diagnosis and treatment for HBV is not performed in Mozambique, since 2002, the country has implemented HBV vaccination in children with an estimated a coverage of 88% [11]. Further screening from donated blood became mandatory in 2006, and in 2024, the strategy to prevent mother-to-child HBV transmission in some health facilities has been implemented [7,12].

HBV genotypes have an important role in controlling the disease in terms of diagnostics, treatment, and predicting disease progression to liver disease [13]. Studies have shown that certain HBV genotypes can influence the clinical evolution of the disease and the treatment response [14]. The severity of liver disease, such as cirrhosis and hepatocellular carcinoma, has been associated with certain HBV genotypes [15]. HBV resistance to antivirals is another factor contributing to treatment failure [16], and the accuracy of laboratory diagnosis can also be affected by mutations in the genome [17].

Studies conducted in Mozambique among blood donors and patients co-infected with HIV (with or without treatment) show that genotype A1 circulates more (around 95%) when compared to genotype E to a lesser extent [18]. Mathew et al. reported the recombination between the E/A genotype in a study of blood donors in Beira (Central Mozambique) [19], and other studies among patients on antiretroviral treatment in the north of Mozambique have reported resistance to lamivudine (3TC) and potential resistance to entecavir (ETV) [20].

Given the limited information of epidemiological data on HBV, this study describes the serological and molecular characteristics within HBV-positive samples from blood donors at the *Hospital Central de Maputo* (HCM) Blood Bank (southern Mozambique).

## 2. Materials and Methods

### 2.1. Study Design and Population

A cross-sectional study was conducted between November 2014 to October 2015 at the HCM. We consecutively included replacement and volunteer blood donors who were present at the blood bank, in accordance with national blood donation standards. Eligible donors included all of those who are at no risk of carrying blood-borne infections (the risk is assessed by interview), aged between 16 and 65, with a weight > 50 kg, body temperature ≤ 37.5 °C, hemoglobin levels of ≥12.0 g/dL for men and ≥11.0 g/dL for women, systolic blood pressure ≤180 mmHg and diastolic blood pressure ≤100 mmHg, no aspirin intake within the last 24 h, no illicit drug use, and no blood transfusions received in the past 12 months. For this study, we selected all the samples confirmed as positive, based on the Enzyme-Linked Immunosorbent Assay (ELISA) and rapid tests described in Section 2.3.

### 2.2. Data Collection

Demographic information (age, sex, nationality, marital status, professional cadre, and others) was obtained from all consenting blood donors using a structured questionnaire. For each participant, a total of 9.0 mL of whole blood was collected into three tubes of 3.0 mL vacutainers with a K3EDTA (Becton Dickinson, Franklin Lakes, NJ, USA). The whole blood was sent to *Instituto Nacional de Saúde* (INS) of Mozambique, where it was centrifuged at 3500 RPM for 10 min to obtain the plasma. The plasma was stored in cryovials at −80 °C until the date of serological and molecular testing.

### 2.3. Serological Assays

Serological testing was performed at the HCM blood bank following the national algorithm, which included: HBsAg screening in plasma samples using the Advanced Quality HBsAg ELISA test kit (InTec Products, INC, China) with 99.9% sensitivity and 100% specificity, and only reactive samples were further confirmed using the Advanced Quality HBsAg Rapid Test (InTec Products, INC, Xiamen, China) with 100% sensitivity and specificity. Non-reactive samples in the first test were considered negative, while those reactive in both tests were considered positive, discordant results were classified as indeterminate and excluded from subsequent steps.

All HBV-positive samples (HBsAg-positive) were tested for anti-HBc, using Bioelisa kits (Biokit, Barcelona, Spain) and HBeAg, using Liaison kits (DiaSorin, Vercelli, Italy), at the INS.

### 2.4. DNA Quantification and Detection

HBV viral load (HBV DNA) was performed from all positive samples using the COBAS AmpliPrep/COBAS TaqMan HBV, v2.0 test (Roche Molecular Systems, Inc., Branchburg, NJ, USA) oche Diagnostics, Germany), with a detection limit of 20 IU/mL.

HBV DNA was extracted from 200 µL of plasma using the High Pure Viral Nucleic Acid Kit (Roche Applied Science, Mannheim, Germany) for all samples with positive results in serological testing. To amplify the S and P regions, primers S1 (position 124–143) and 4R (position 1120–1100) were used in the first round, and primers 1F (position 180–203) and 4R were used in the second round. The PCR was conducted with 30 cycles: at an initial denaturation at 94 °C for 1 min, followed by 94 °C for 15 s, 56 °C for 30 s, 68 °C for 1 min and 15 s, and a final extension at 68 °C for 10 min. The amplified product was visualized on a 2% agarose gel stained with ethidium bromide under ultraviolet light and purified using the High Pure PCR Product Purification Kit (Roche). The PCR products (~900 bases) were sequenced using a 3500 Genetic Analyzer (Foster City, CA, USA).

### 2.5. Genetic Analysis of HBV S Gene

A total of 256 HBV whole-genome reference sequences (WGS), representing genotypes A and E, with at least two sequences for each genotype, were retrieved from GenBank and combined with the region S sequences from the current study. Sequences with incomplete metadata (i.e., collection date or origin) were excluded, reducing the dataset to 109 HBV sequences. All 109 sequences were aligned using MAFFT with the FFT-NS-2 algorithm and the default guide tree based on the modified UPGMA algorithm [21]. A phylogenetic analysis was conducted using RAxML under the GTR+F+I+G4 nucleotide substitution model, selected as the best-fit model based on the Bayesian Information Criterion (BIC) in the jModelTest 2 program. Random starting trees were employed, and a total of 1000 rapid bootstrap replicates were performed with all other parameters set to default. The resulting tree was visualized and analyzed using the ggtree package implemented in R. The tree was rooted with an outgroup sequence from genotype I of Vietnam. Additionally, BioEdit v7.2.5 [22] was utilized for a serotypic analysis of the HBV study sequences. This was performed by comparing amino acid residues at positions 122, 127, 134, 159, and 160 of the HBV S gene sequences with the reference sequence X02763.1

Genotype analyses were performed using the Hepatitis B Virus Database (HBVDB) tool to ensure the accurate identification of the genotypes. Subsequently, drug resistance analyses were conducted using the Stanford HBVSeq platform (Stanford HBVSeq). The analyses were complemented by the HBV Geno2Pheno version 2 tool (HBV Geno2Pheno) [23].

### 2.6. Data Management and Statistical Analysis

The data were organized in a Microsoft Excel spreadsheet and statistically analyzed using SPSS software version 26.0. The frequencies of categorical variables, such as HBV genotypes, were calculated and presented in tables. The association between categorical variables was assessed using Fisher’s exact test and Pearson’s Chi-square test, with *p* < 0.05 considered the threshold for statistical significance.

## 3. Results

### 3.1. Sample Testing and Sociodemographic Characteristics

**Figure 1:**
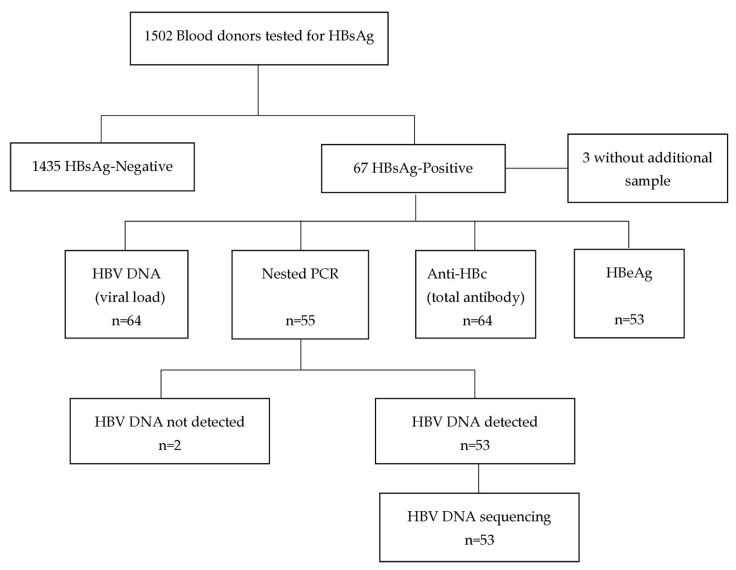
Flowchart of screening and characterization of positive hepatitis B virus (HBV) samples in Blood donors in Maputo City.

Of a total of 1502 blood donors, 67 were positive for HBV with an estimated frequency of 4.4% and 53 were successfully sequenced as summarized in Figure 1. Among the HBV-positive blood donors, the majority were male with 79.1% (n = 53) and aged between 25 and 39 years with 55.2% (n = 37), as shown in Table 1. Most of the HBV-positive blood donors had completed high school with 65.7% (n = 44), were single with 73.1% (n = 49), and were replacement donors with 89.6% (n = 60).

### 3.2. HBV Serological and Virological Markers

From the HBsAg-positive blood donors, 96.9% (62/64) were positive for Anti-HBc and 5.7% (3/53) were positive for HBeAg (Table 2). The viral load of the two samples negative for Anti-HBc was 1977 and 721 IU/mL, respectively. Two of the HBeAg-positive samples had a viral load above the quantification limit (>170,000,000 IU/mL) and one had a viral load of 10,284 IU/mL.

The median viral load of HBV-positive blood donors was 1288.5 IU/mL, and 43.8% (28) had a viral load higher than 2000 IU/mL. Three sample had a viral load >170,000,000 IU/mL.

### 3.3. Genetic Analysis of the S Region

**Figure 2:**
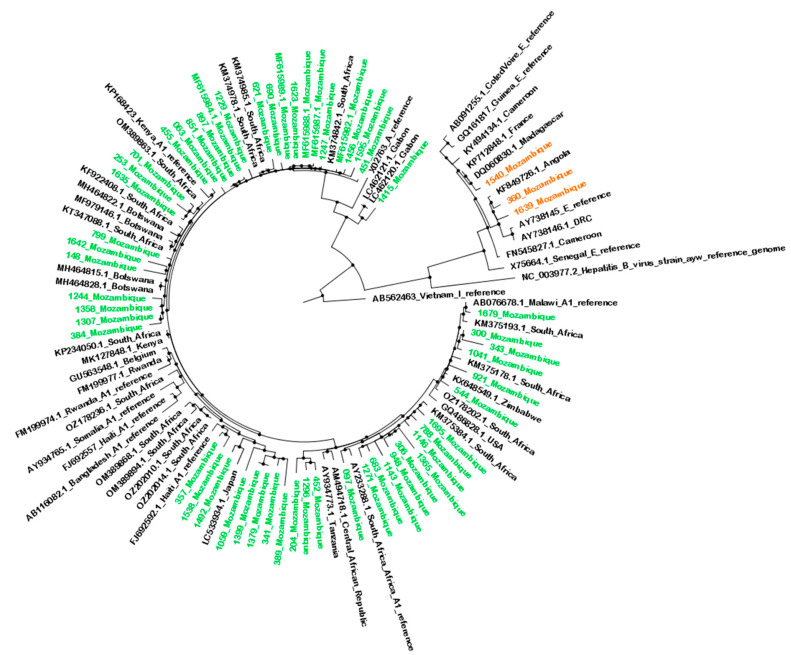
Maximum likelihood phylogenetic tree of HBV sequences based on the alignment of 53 Mozambican sequences (S region) alongside 56 reference sequences of HBV genotypes. Mozambican sequences are highlighted: green for subgenotype A1 and orange for genotype E. Reference sequences are represented by their accession numbers and country of origin. The scale bar indicates the number of substitutions per site.

From the samples sequenced, 94.3% (n = 50) were classified as genotype A1 and 5.7% (n = 3) as genotype E (Table 2). Figure 2 shows the similarity between the 53 sequences in this study and other sequences from Mozambique and other parts of the world.

**Figure 3:**
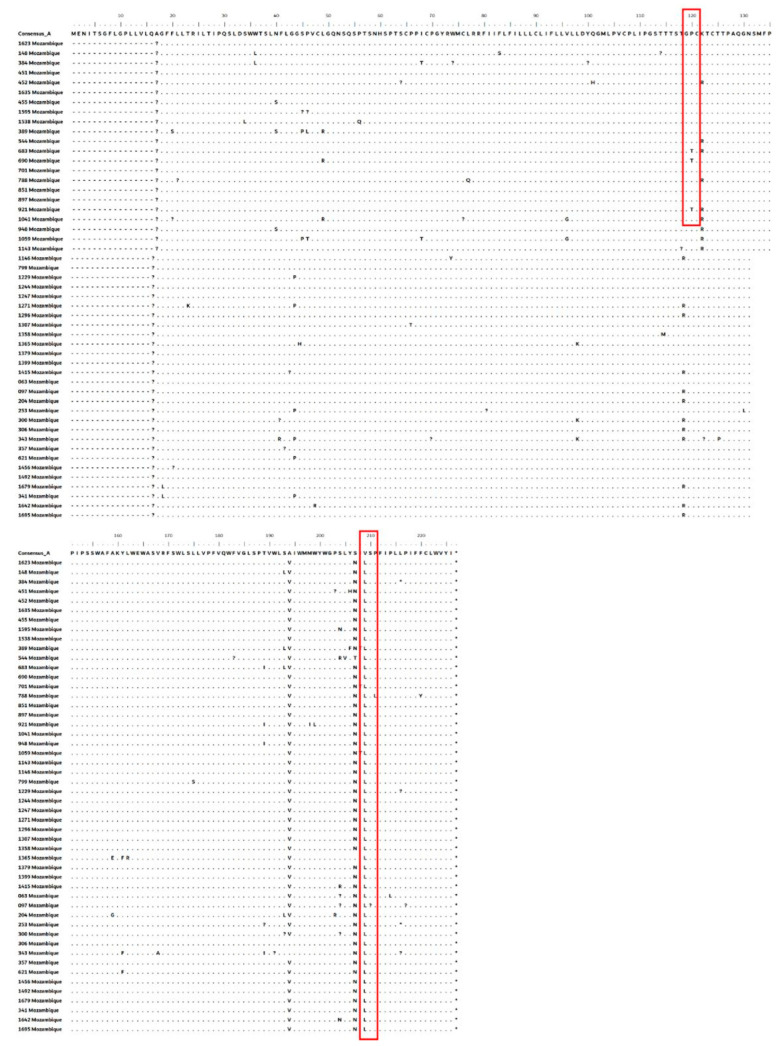
Amino acid alignment of the S gene (aa 1–227) from occult hepatitis B virus (HBV)-infected individuals with genotype A from Mozambique.

**Figure 4:**
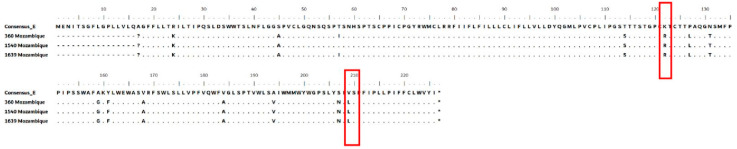
Amino acid alignment of the S gene (aa 1–227) from occult hepatitis B virus (HBV)-infected individuals with genotype E from Mozambique.

The presence of amino acid substitutions at different positions in the surface region was observed in both genotypes A and E of HBV sequences. The substitution V209L was observed in all HBV genotype A sequences (Figure 3 and Figure 4). Our study showed escape mutations within the major hydrophilic region (MHR) of HBsAg in the second loop of “a” determinant associated with vaccine-induced immunity in several HBV isolates (Figure 3). The substitutions at position 129 (Q129P) were observed in only one sample (Figure 3). Mutations outside the ‘‘a’’ determinant were located mainly at positions 120 and 123 included P120R only among the genotype A1 samples and K123R in both A1 and E samples.

## 4. Discussion

HBV infection remains a major public health threat, particularly for blood safety. In our study, the high frequency of HBV infection in replacement donors, young people, and males is in concordance with other studies carried out in Mozambique and Africa [7,24,25].

We also detected a high frequency of anti-HBc (96.9%) among the HBV-positive samples, but a much lower frequency of HBeAg was detected in three samples where two had a viral load above the detection limit. Such findings are not surprising since a lower frequency of HBeAg was found to be common among individuals infected with genotype A1 [26,27,28], which is also predominant in Mozambique.

Alternatively, in the absence of an HBV viral load test, the use of HBeAg is recommended to monitor viral replication [29]. Nevertheless, our results show that most of the samples with viral load >2000 were negative for HBeAg. Such results just reinforce the need to use other markers instead as the criteria to initiate HBV treatment. Such may include the use of APRI (aspartate aminotransferase-to-platelet ratio index), FIB-4 (Fibrosis-4 score), intracellular enzymes (alanine aminotransferase -ALT and aspartate aminotransferase-AST) or transient elastography, previously shown to be effective as non-invasive tests to access the degree of necroinflammation and fibrosis and can be used in countries with limited resources and no capacity for routine HBV viral load testing [30,31,32].

Half of the HBV-positive blood donors had a viral load result > 2000, and according to the guidelines [32], they are eligible to initiate HBV treatment. The percentage of HBV diagnosis and treatment in the general population in many countries in Africa is low or non-existent [33]. Notification and clinical follow-up of HBV-positive blood donors is not only important for the prevention of transfusion-transmitted infections but can also contribute to the elimination of Hepatitis B as a public health problem [34]. Almost all African countries screen blood donors for HBV, but those who test positive are not followed up clinically [35,36].

With regard to the genotypes, A1 was observed at a higher frequency and genotype E at a lower. Our results are similar to Chambal et al. and Mabunda et al., who carried out studies in Mozambique [18,26] and in other African countries [37]. Studies in other areas of the country and with other populations may yield different results from those found by us. For example, studies that looked into HBV genetic diversity in the central and north region of Mozambique showed a higher diversity when compared to south region [19,20]. On the other hand, factors such as age, gender, and race can also influence genetic diversity and the serological profile [38,39].

Additionally, the risk of developing HCC is 4.5 times higher in participants infected with genotype A1 when compared to those with other genotypes [40,41]. This may explain the high incidence of HCC in Mozambique [4,42]. Moreover, no major mutations associated with resistance to antivirals used to treat HBV were observed, which makes it easier to implement better treatment strategies if such cases among blood are properly diagnosed. These results are similar to the results from neighboring countries conducted among blood donors [36,43,44].

The V209L and A194V substitution found in all the genotype A1 sequences in this study was also found by Mabunda et al. in blood donors in the city of Maputo [26], which might suggest a molecular signature for these samples in which the impact is still unknown. Other studies carried out in African populations have found substitution, but without describing its impact on the dynamics of HBV infection [45]. In addition, the Q129P substitutions have been reported in other studies and are associated with antigenicity prediction [46].

Frequent substitutions at positions P120T, potentially alter the epitope conformation and affect its antigenicity. Position 120 allows substitutions to Gly/Thr/Ser/Asn/Gln, while it is associated with vaccine escape [47]. Raheel et al. observed polymorphism P120T mostly in genotype D, while in our study, it was common in A1. This just emphasizes the occurrence of such polymorphisms in different geographical regions [46] and other genotypes. A study conducted in China detected the K122R substitution in patients with acute hepatitis [48], which was also reported here in high frequency and can also be associated with the high viral load observed.

Few studies in Mozambique have investigated mutations associated with drug resistance or vaccine escape mutations, with only one study identifying mutations to date [20]. However, these mutations were not detected in this study, either in blood donors from Beira or Maputo [19,26].

The implementation of strategies to eliminate viral hepatitis as a public health problem remains below expectations in African countries [49,50]. The opportunity for accurate diagnostic tools and adequate treatment among HBV-positive blood donors in many African countries is still underestimated and requires attention. Such improvements include better health service organizations particularly among HIV-positive patients, more investment in HBV treatment, global solidarity, and the commitment of local leaders.

Our study also presents some limitations: (a) The limited number of sequences generated and only from one region may not truly reflect the real disease burden. However, this preliminary result among our population can serve as a guideline to suggest better public health policies for HBV diagnosis and treatment. (b) The lack of testing for all serological markers in HBV-positive donors, as well as the absence of such testing in HBV-negative donors, did not allow for the assessment of immunization status, susceptibility to infection, and the phase of HBV infection.

## 5. Conclusions

Understanding the molecular epidemiology and the clinical implication associated with the HBV disease burden is important. Our results no doubt emphasize the need for better diagnostic strategies for HBV among blood donors. A high positivity rate was observed among blood donors with high viral loads, and if not diagnosed and treated, the risk of progressing to live diseases is higher. Further, genotype A1 predominated among our blood donors, and no mutations were associated with drug resistance, putting them in a good position to initiate treatment successfully. These findings highlight the need for continuous surveillance to guide effective HBV control strategies and the opportunity for clinical follow-up of HBV-positive patients screened in blood banks in Mozambique and other African countries.

## Figures and Tables

**Table 1:** Sociodemographic characteristics of the 67 HBsAg-positive blood donors.

Sociodemographic Characteristics		HBsAg-Positive
		n (%)
Gender	Male	53 (79.1)
	Female	14 (20.9)
Age Range (years)	15 a 24 years	19 (28.4)
	25 a 39 years	37 (55.2)
	40 a 59 years	11 (16.4)
Education Level	No schooling	1 (1.5)
	Primary	17 25.4)
	Secondary/Technical	44 (65.7)
	Higher	9 (13.4)
Marital Status	Single	49 (73.1)
	Married	17 (25.4)
	Divorced	1 (1.5)
Donor Type	Replacement	60 (89.6)
	Voluntary	7 (10.4)
Employment	Student	19 (28.4)
	Formal employment	21 (31.3)
	Informal employment	27 (40.3)

**Table 2:** Serological and molecular characteristics of HBV-positive samples in blood donors at the blood bank of Maputo Central Hospital.

Serological Markers	n (%)
**Anti-HBc (n)**	(n = 64)
Positive	62 (96.9)
Negative	2 (3.1)
**HBeAg (n)**	(n = 53)
Positive	3 (5.7)
Negative	50 (94.3)
**Virological markers**	n (%)
**Genotypes**	(n = 53)
A1	50 (94.3%)
E	3 (5.7%)
**Viral Load (IU/mL)**	(n = 64)
Viral load Median (Min–Max)	1288.5 (<20 to >170,000,000)
Viral load < 2000	36 (56.2%)
Viral load > 2000	28 (43,8%)

## Data Availability

Data are contained within the article.

## References

[1] Iacobucci G. (2024). Hepatitis: WHO Publishes Warning over Rise in Global Deaths. BMJ.

[2] World Health Organizacion (2024). Global Hepatitis Report 2024: Action for Access in Low- and Middle-Income Countries.

[3] Jayalakshmi M., Kalyanaraman N., Pitchapp R., Rosas-Acosta G. (2013). Hepatitis B Virus Genetic Diversity: Disease Pathogenesis. Viral Replication.

[4] Kew M.C. (2013). Epidemiology of Hepatocellular Carcinoma in Sub-Saharan Africa. Ann. Hepatol..

[5] Carrilho C., Fontes F., Tulsidás S., Lorenzoni C., Ferro J., Brandão M., Ferro A., Lunet N. (2019). Cancer Incidence in Mozambique in 2015–2016: Data from the Maputo Central Hospital Cancer Registry. Eur. J. Cancer Prev..

[6] Viegas E.O., Tembe N., Macovela E., Gonçalves E., Augusto O., Ismael N., Sitoe N., De Schacht C., Bhatt N., Meggi B. (2015). Incidence of HIV and the Prevalence of HIV, Hepatitis B and Syphilis among Youths in Maputo, Mozambique: A Cohort Study. PLoS ONE.

[7] Mabunda N., Augusto O., Zicai A.F., Duajá A., Oficiano S., Ismael N., Vubil A., Mussá T., Moraes M., Jani I. (2022). Nucleic Acid Testing Identifies High Prevalence of Blood Borne Viruses among Approved Blood Donors in Mozambique. PLoS ONE.

[8] Revill P.A., Chisari F.V., Block J.M., Dandri M., Gehring A.J., Guo H., Hu J., Kramvis A., Lampertico P., Janssen H.L.A. (2019). A Global Scientific Strategy to Cure Hepatitis B. Lancet Gastroenterol. Hepatol..

[9] Wong G.L.-H., Wong V.W.-S. (2016). Eliminating Hepatitis B Virus as a Global Health Threat. Lancet Infect. Dis..

[10] Howell J., Seaman C., Wallace J., Xiao Y., Scott N., Davies J., De Santis T., Adda D., El-Sayed M., Feld J.J. (2023). Pathway to Global Elimination of Hepatitis B: HBV Cure Is Just the First Step. Hepatology.

[11] Cassocera M., Chissaque A., Martins M.R.O., Deus N.D. (2020). 40 Years of Immunization in Mozambique: A Narrative Review of Literature, Accomplishments, and Perspectives. Cad. Saúde Pública.

[12] Loarec A., Nguyen A., Molfino L., Chissano M., Madeira N., Rusch B., Staderini N., Couto A., Ciglenecki I., Tamayo Antabak N. (2022). Prevention of Mother-to-Child Transmission of Hepatitis B Virus in Antenatal Care and Maternity Services, Mozambique. Bull. World Health Org..

[13] Lin C., Kao J. (2011). The Clinical Implications of Hepatitis B Virus Genotype: Recent Advances. J. Gastroenterol. Hepatol..

[14] Cao G.-W. (2009). Clinical Relevance and Public Health Signifcance of hepatitisB Virus Genomic Variations. WJG.

[15] Tong S., Revill P. (2016). Overview of Hepatitis B Viral Replication and Genetic Variability. J. Hepatol..

[16] Locarnini S. (2008). Primary Resistance, Multidrug Resistance, and Cross-Resistance Pathways in HBV as a Consequence of Treatment Failure. Hepatol. Int..

[17] Guvenir M., Arikan A. (2020). Hepatitis B Virus: From Diagnosis to Treatment. Pol. J. Microbiol..

[18] Chambal L.M., Samo Gudo E., Carimo A., Corte Real R., Mabunda N., Maueia C., Vubil A., Zicai A.F., Bhatt N., Antunes F. (2017). HBV Infection in Untreated HIV-Infected Adults in Maputo, Mozambique. PLoS ONE.

[19] Mathew A., Ismael N., Meeds H., Vubil A., Zicai A.F., Mabunda N., Blackard J.T. (2023). Hepatitis B Virus Genotypes and Drug Resistance Mutations Circulating in Blood Donors in Beira, Mozambique. PLoS ONE.

[20] Wandeler G., Musukuma K., Zürcher S., Vinikoor M.J., Llenas-García J., Aly M.M., Mulenga L., Chi B.H., Ehmer J., Hobbins M.A. (2016). Hepatitis B Infection, Viral Load and Resistance in HIV-Infected Patients in Mozambique and Zambia. PLoS ONE.

[21] Katoh K., Rozewicki J., Yamada K.D. (2019). MAFFT Online Service: Multiple Sequence Alignment, Interactive Sequence Choice and Visualization. Brief. Bioinform..

[22] https://www.sciencedirect.com/science/article/abs/pii/S0006291X24016978.

[23] Analysis of the Complete Genome of Hepatitis B… | F1000Research. https://f1000research.com/articles/7-1023/v3?s3BucketUrl=https%3A%2F%2Ff1000research.s3.amazonaws.com&gtmKey=GTM-PCBS9JK&submissionUrl=%2Ffor-authors%2Fpublish-your-research&otid=1bc074d1-3db4-47ed-9f80-df1a4a3f2ab4&immUserUrl=https%3A%2F%2Ff1r-proxy.f1krdev.com%2Feditor%2Fmember%2Fshow%2F.

[24] Cunha L., Plouzeau C., Ingrand P., Gudo J.P.S., Ingrand I., Mondlane J., Beauchant M., Agius G. (2007). Use of Replacement Blood Donors to Study the Epidemiology of Major Blood-borne Viruses in the General Population of Maputo, Mozambique. J. Med. Virol..

[25] Stokx J., Gillet P., De Weggheleire A., Casas E.C., Maendaenda R., Beulane A.J., Jani I.V., Kidane S., Mosse C.D., Jacobs J. (2011). Seroprevalence of Transfusion-Transmissible Infections and Evaluation of the Pre-Donation Screening Performance at the Provincial Hospital of Tete, Mozambique. BMC Infect. Dis..

[26] Mabunda N., Zicai A.F., Ismael N., Vubil A., Mello F., Blackard J.T., Lago B., Duarte V., Moraes M., Lewis L. (2020). Molecular and Serological Characterization of Occult Hepatitis B among Blood Donors in Maputo, Mozambique. Mem. Inst. Oswaldo Cruz.

[27] Tonetto P.A., Gonçales N.S., Fais V.C., Vigani A.G., Gonçales E.S., Feltrin A., Gonçales F.L. (2009). Hepatitis B Virus: Molecular Genotypes and HBeAg Serological Status among HBV-Infected Patients in the Southeast of Brazil. BMC Infect. Dis..

[28] Ambachew H., Zheng M., Pappoe F., Shen J., Xu Y. (2018). Genotyping and Sero-Virological Characterization of Hepatitis B Virus (HBV) in Blood Donors, Southern Ethiopia. PLoS ONE.

[29] Li Y., Zhu Y., Gao D., Pan Y., Wang J., Zhang S., Yan X., Zhu L., Zhu C., Liu X. (2024). HBeAg-Positive CHB Patients with Indeterminate Phase Associated with a High Risk of Significant Fibrosis. Virol. J..

[30] Wang H., Xue L., Yan R., Zhou Y., Wang M.S., Cheng M.J., Huang H.J. (2013). Comparison of FIB-4 and APRI in Chinese HBV-Infected Patients with Persistently Normal ALT and Mildly Elevated ALT. J. Viral Hepat..

[31] Zhang T., Liu Z., Zhao X., Mao Z., Bai L. (2019). A Novel Prognostic Score Model Based on Combining Systemic and Hepatic Inflammation Markers in the Prognosis of HBV-Associated Hepatocellular Carcinoma Patients. Artif. Cells Nanomed. Biotechnol..

[32] Guidelines for the Prevention, Diagnosis, Care and Treatment for People with Chronic Hepatitis B Infection. https://www.who.int/publications/i/item/9789240090903.

[33] Fofana D.B., Somboro A.M., Maiga M., Kampo M.I., Diakité B., Cissoko Y., McFall S.M., Hawkins C.A., Maiga A.I., Sylla M. (2023). Hepatitis B Virus inWest African Children: Systematic Review and Meta-Analysis ofHIV and Other Factors Associated with Hepatitis BInfection. Int. J. Environ. Res. Public Health.

[34] Spearman C.W., Afihene M., Ally R., Apica B., Awuku Y., Cunha L., Dusheiko G., Gogela N., Kassianides C., Kew M. (2017). Hepatitis B in Sub-Saharan Africa: Strategies to Achieve the 2030 Elimination Targets. Lancet Gastroenterol. Hepatol..

[35] Quintas A.E., Cuboia N., Cordeiro L., Sarmento A., Azevedo L. (2024). Seroprevalence of Hepatitis B Virus Surface Antigen among African Blood Donors: A Systematic Review and Meta-Analysis. Front. Public Health.

[36] Mohamed Z., Kim J.U., Magesa A., Kasubi M., Feldman S.F., Chevaliez S., Mwakale P., Taylor-Robinson S.D., Thursz M.R., Shimakawa Y. (2019). High Prevalence and Poor Linkage to Care of Transfusion-transmitted Infections among Blood Donors in Dar-es-Salaam, Tanzania. J. Viral Hepat..

[37] Kafeero H.M., Ndagire D., Ocama P., Kato C.D., Wampande E., Walusansa A., Kajumbula H., Kateete D., Ssenku J.E., Sendagire H. (2023). Mapping Hepatitis B Virus Genotypes on the African Continent from 1997 to 2021: A Systematic Review with Meta-Analysis. Sci. Rep..

[38] Lau D.T.Y., Ganova-Raeva L., Wang J., Mogul D., Chung R.T., Lisker-Melman M., Chang K.-M., Shaikh O.S., Janssen H.L.A., Wahed A.S. (2021). Precore and Basal Core Promoter Hepatitis B Virus (HBV) Variants Are Present From a Young Age and Differ Across HBV Genotypes. Hepatology.

[39] Di Bisceglie A.M., King W.C., Lisker-Melman M., Khalili M., Belle S.H., Feld J.J., Ghany M.G., Janssen H.L.A., Lau D., Lee W.M. (2019). Age, Race and Viral Genotype Are Associated with the Prevalence of Hepatitis B e Antigen in Children and Adults with Chronic Hepatitis B. J. Viral Hepat..

[40] Kramvis A., Kew M.C. (2007). Epidemiology of Hepatitis B Virus in Africa, Its Genotypes and Clinical Associations of Genotypes. Hepatol. Res..

[41] Kew M.C., Kramvis A., Yu M.C., Arakawa K., Hodkinson J. (2005). Increased Hepatocarcinogenic Potential of Hepatitis B Virus Genotype A in Bantu-speaking Sub-saharan Africans. J. Med. Virol..

[42] Cunha L., Carrilho C., Bhatt N., Loforte M., Maueia C., Fernandes F., Guisseve A., Mbofana F., Maibaze F., Mondlane L. (2019). Hepatocellular Carcinoma: Clinical-Pathological Features and HIV Infection in Mozambican Patients. Cancer Treat. Res. Commun..

[43] Choga W.T., Anderson M., Zumbika E., Moyo S., Mbangiwa T., Phinius B.B., Melamu P., Kayembe M.K., Kasvosve I., Sebunya T.K. (2019). Molecular Characterization of Hepatitis B Virus in Blood Donors in Botswana. Virus Genes.

[44] Singogo E., Chagomerana M., Van Ryn C., M’bwana R., Likaka A., M’baya B., Puerto-Meredith S., Chipeta E., Mwapasa V., Muula A. (2023). Prevalence and Incidence of Transfusion-Transmissible Infections among Blood Donors in Malawi: A Population-Level Study. Transfus. Med..

[45] Mokaya J., McNaughton A.L., Hadley M.J., Beloukas A., Geretti A.-M., Goedhals D., Matthews P.C. (2018). A Systematic Review of Hepatitis B Virus (HBV) Drug and Vaccine Escape Mutations in Africa: A Call for Urgent Action. PLoS Negl. Trop. Dis..

[46] Raheel M., Choga W.T., Blackard J.T. (2020). The Distribution of Hepatitis B Virus Surface Antigen Polymorphisms at Positions Associated with Vaccine Escape. J. Med. Virol..

[47] Ma Q., Wang Y. (2012). Comprehensive Analysis of the Prevalence of Hepatitis B Virus Escape Mutations in the Major Hydrophilic Region of Surface Antigen. J. Med. Virol..

[48] Wang J., Liu Y., Liao H., Liu L., Chen R., Si L., Luo D., Huang B., Li L., Jiang J. (2020). The sK122R Mutation of Hepatitis B Virus (HBV) Is Associated with Occult HBV Infection: Analysis of a Large Cohort of Chinese Patients. J. Clin. Virol..

[49] McNaughton A.L., Lourenço J., Bester P.A., Mokaya J., Lumley S.F., Obolski U., Forde D., Maponga T.G., Katumba K.R., Goedhals D. (2020). Hepatitis B Virus Seroepidemiology Data for Africa: Modelling Intervention Strategies Based on a Systematic Review and Meta-Analysis. PLoS Med..

[50] Torimiro J.N.E., Duri K., Goumkwa N.M., Atah S.M., Ndzie Ondigui J.-L., Lobe C., Bouyou M., Ndeboko B., Mahamat Moussa A., Police C. (2024). Toward the Elimination of Hepatitis B: Networking to Promote the Prevention of Vertical Transmission of Hepatitis B Virus through Population-Based Interventions and Multidisciplinary Groups in Africa. Front. Public Health.

